# 11p15.4 Microdeletion Associates with Hemihypertrophy

**DOI:** 10.1155/2018/2746347

**Published:** 2018-10-30

**Authors:** Surasak Puvabanditsin, Mehrin Sadiq, Marianne Jacob, Maaz Jalil, Kenya Cabrera, Omer Choudry, Rajeev Mehta

**Affiliations:** Department of Pediatrics, Rutgers Robert Wood Johnson Medical School, New Brunswick, NJ, USA

## Abstract

We report a preterm female infant with intrauterine growth retardation, dysmorphic facies, missing rib, small hands and feet, and hemihypertrophy. The results of whole genome SNP microarray analysis showed approximately 77 Kb interstitial deletion of the short arm of chromosome 11 (11p15.4). We report novel clinical findings of this rare genetic condition.

## 1. Introduction

Microdeletion of the short arm of chromosome 11 is a rare chromosomal anomaly. The “pure” deletion of 11p15 region with no other chromosomal imbalance is extremely uncommon. Beckwith-Wiedemann syndrome (BWS)/overgrowth syndrome is known to be associated with genetic and/or epigenetic alterations that modify imprinted gene expression on chromosome 11p15.5 [1–5]. The phenotypes associated with microdeletion 11p15.4 have not been previously reported.* The deletion in this region in relation to hemihypertrophy (overgrowth syndrome) is novel*. We report a novel case of 11p15.4 deletion and a review of the literature.

## 2. Case Presentation

**Figure 1:**
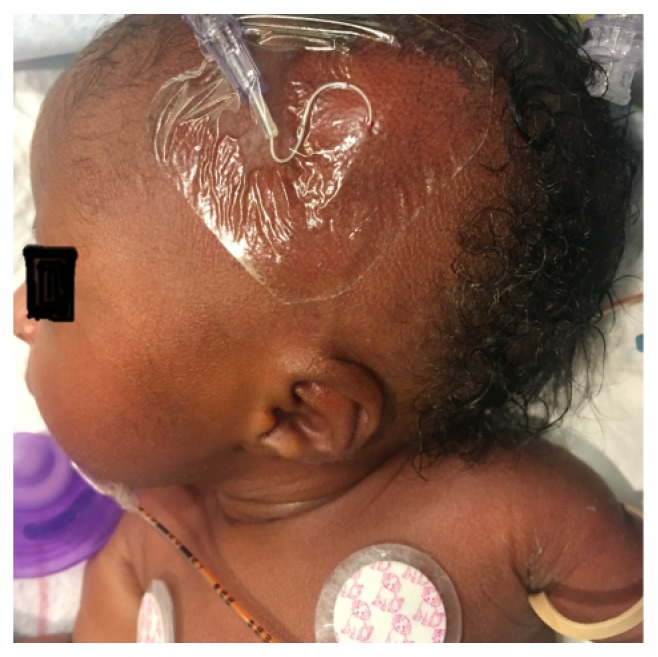
Photo showed low-set and posteriorly rotated ear.

**Figure 2:**
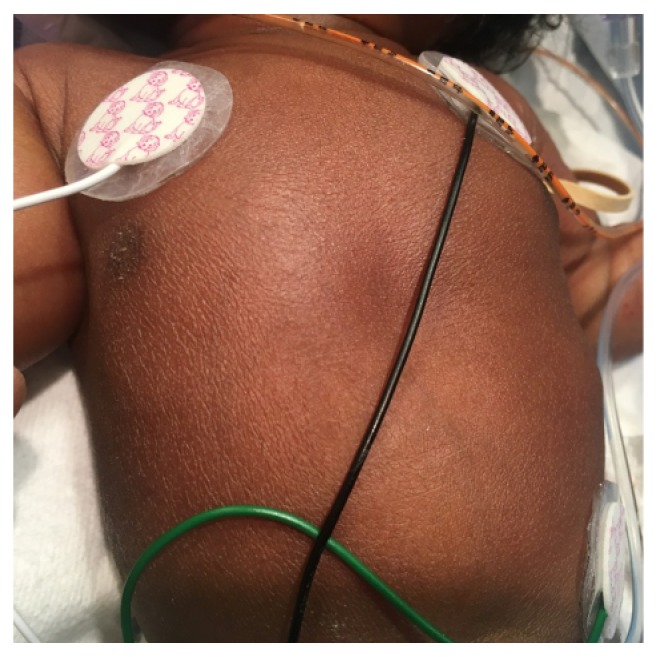
Photo showed wide-spaced nipples.

**Figure 3:**
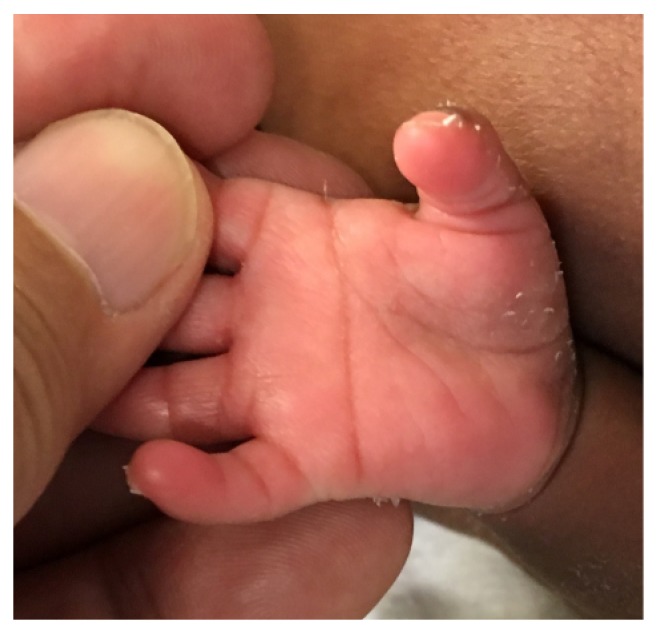
Photo showed palmar crease.

**Figure 4:**
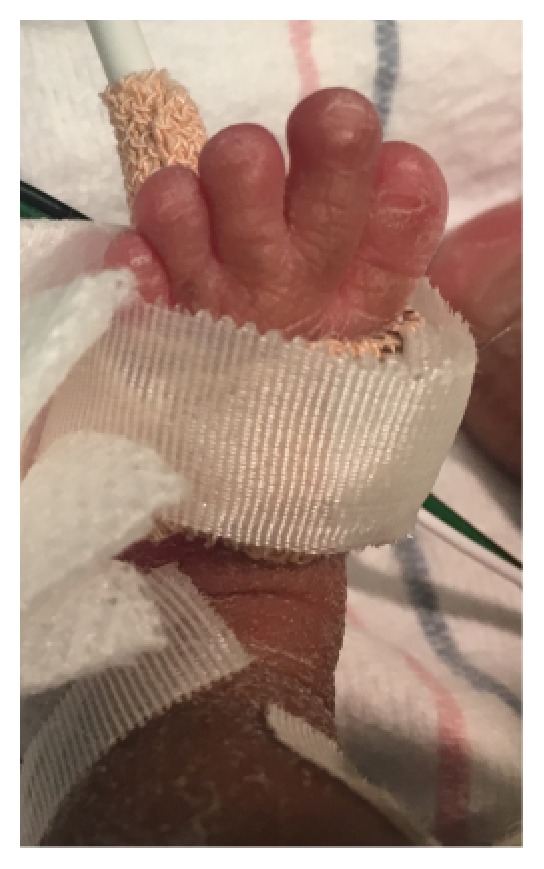
Photo showed overgrowth of the second toe.

**Figure 5:**
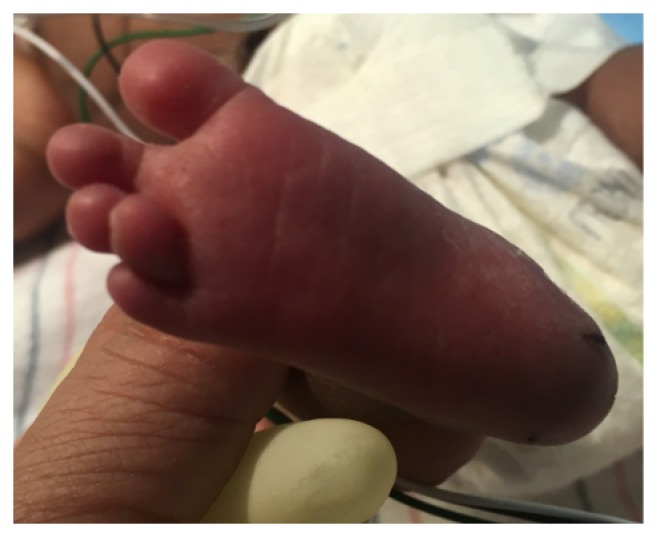
Photo showed overlapping of the third and fourth toe.

**Figure 6:**
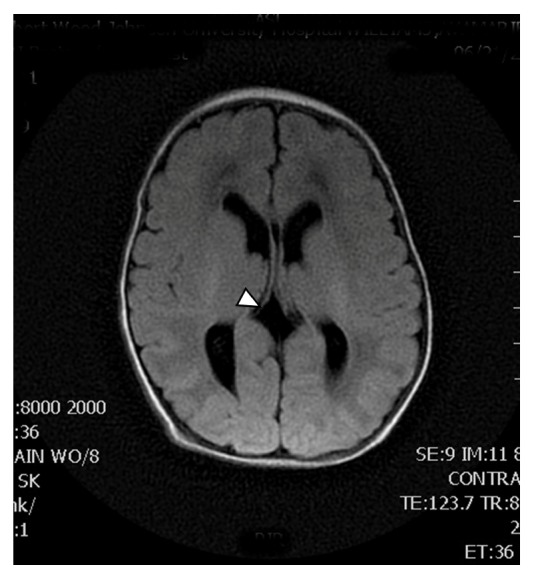
Arachnoid cyst at the velum interpositum (arrow).

**Figure 7:**
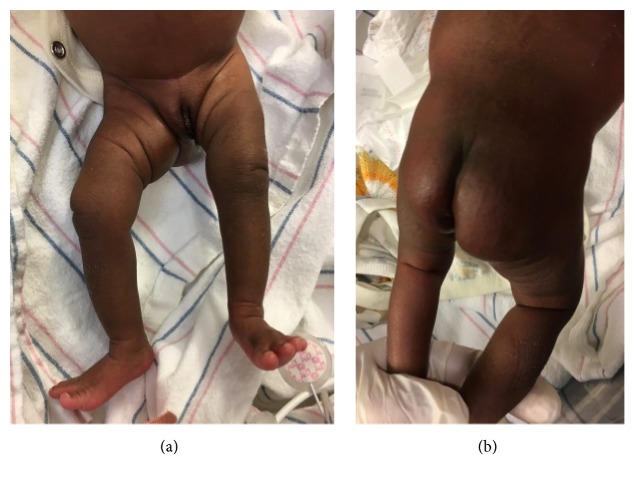
(a) and (b) Photos showed hemihypertrophy.

A 1415-gram female infant was delivered at 34^6/7^ weeks of gestation to a 40-year-old primigravida by cesarean section secondary to preeclampsia and abnormal middle cerebral artery Doppler assessment. Apgar scores were 8 and 9 at 1 and 5 minutes, respectively. Pregnancy was complicated with a diagnosis of severe intrauterine growth retardation. Family history revealed a 7-year-old half-sister with hereditary anemia. There was no in utero exposure to known teratogens. No genetic test was performed during pregnancy. Physical examination revealed a weight of 1415 g (<3rd centile), length 34 cm (<3rd centile), and head circumference 29 cm (5th centile). He was noted to have downslanted palpebral fissures, low-set and posteriorly rotated ears, wide space nipples, palmar crease, small hands and feet, rocker bottom feet, overgrowth 2nd toes, and overlapping 3rd and 4th toes (Figures 1, 2, 3, 4, and 5). Chest radiography revealed 11 ribs. Cranial MRI scan showed mildly dilated lateral and third ventricles, and there was a 17x13 mm arachnoid cyst at the velum interpositum (Figure 6). Echocardiogram revealed left-side aortic arch. Genetic testing was performed at 4 days of age. During 4 weeks of hospitalization, asymmetrical growth of left and right sides of the body and extremities was noted (Figure 7). The infant's blood count and red cell indices (mean corpuscular volume) were unremarkable. She was discharged home at 31 days of age.

**Figure 8:**
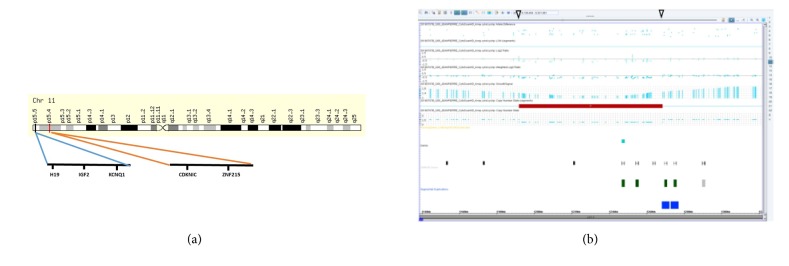
(a) Ideogram of chromosome 11 and deleted region (red bar) and genes involved with BWS. (b) Microarray showed 11p15.4 deletion (arrows).

On the blood sample that was collected for genetic testing on day 4 of life, whole genome SNP (Single Nucleotide Polymorphisms) microarray analysis was performed using the Affymetrix CytoScan HD platform which uses over 743,000 SNP probes and 1,953,000 NPCN probes with median spacing of 0.88 kb. 250 ng of total genomic DNA extracted from lymphocytes was digested with NspI and then ligated to NspI adaptors, respectively, and amplified using Titanium Taq with a GeneAmp PCR system 9700. There was a 77 kilobase (kb) microdeletion at 11p15.4 arr [hg19] 11p15.4(5,191,871-5,268,465) x 1(Figure 8). The deleted region includes 3 OMIM genes (*HBB, HBD, *and* BGLT3*).

## 3. Discussion

The clinical features of Beckwith-Wiedemann syndrome (BWS) include hemihypertrophy and/or macroglossia. Hypoglycemia is reported in 30-50% of the babies with BWS [1].

The genetics of BWS is complex and involves multiple genes on chromosome 11p15 [2–6]. Three regions on chromosome 11p15* (BWSCR1, BWSCR2, and BWSCR3)* may play a role in the development of BWS. The* ZNF214* and* ZNF215* genes are located in one of the three regions on chromosome 11p15, called the Beckwith-Wiedemann syndrome chromosome region-2 (BWSCR2). The* H19 *(103280),* ICR1 *(616186), and* KCNQ1OT1 *(604115) are located at 11p15.5; and the* CDKN1C *(60856) is located at 11p54 [1, 7, 8].

The cyclin-dependent kinase inhibitor 1C (CDKN1C) gene encodes for making a protein that helps regulate growth. This protein acts as a tumor suppressor. It is also involved in controlling fetal growth and stops the developing fetus from becoming too large. The activity of the CDKN1C gene depends on which parent it was inherited from.* Paternally imprinted gene would deactivate the activity of CDKN1C gene [9].*

BWS is a condition that causes overgrowth and other signs and symptoms that affect various parts of the body. More than half of all BWS cases result from alterations in methylation of the imprinting center 2 region (IC2). The maternally inherited copy of the IC2 region shows a decrease in the methylation, and this abnormality adversely affects* the regulation of severa*l genes that are normally controlled by IC2, including CDKN1C. Reduction in the activity this gene, which normally controls cell growth and division, leads to overgrowth and the other features of BWS. Rarely, BWS has been caused by the deletion of a small amount of DNA from the maternally inherited copy of the IC2 region.

Intrauterine growth restriction has been reported to be associated with both paternal and maternal 11p15 abnormalities [9, 10]. At least six mutations in the CDKN1C gene have been found to cause this condition [9].

Deletion of the 11p15.4 region is associated with autosomal dominant hereditary persistence thalassemia (HPFH, OMIM: 141749) and autosomal recessive beta-thalassemia (OMIM: 141900) [11, 12].

We report on a preterm infant who had intrauterine growth retardation and developed hemihypertrophy during the postnatal stay. The microdeletion of 11p15.4 that was found in our case is likely to be paternally derived, since the patient's half-sister had an inherited anemia (family history). The additional features (abnormal physical findings) in our patient have not been previously reported with deletion of the 11p15.4 region. Microdeletions of 11p15 are rare. There have been limited reports of patients with interstitial deletions involving band 11p15.4. To our knowledge none have reported clinical features in a neonate with the same deletion as in our case. Here, we provide additional human genetic evidence that the 11p15.4 deletion contains regulatory elements that play a mechanistic role in the hemihypertrophy BWS phenotype and intrauterine growth retardation (IUGR).* It remains unclear what exactly is the relation between this deletion and the BWS/hemihypertrophy associated genes.*

In summary, we report a novel case of a dysmorphic preterm neonate with* microdeletion* 11p15.4. These findings may help identify the gene implicated in BWS and IUGR.
